# IgE levels correlate with Fc*ε*RI expression on circulatory basophils but not with their activation response in patients with Hymenoptera anaphylaxis

**DOI:** 10.3389/falgy.2026.1864564

**Published:** 2026-07-20

**Authors:** Stefan Aigner, Viktoria Puxkandl, Teresa Burner, Angelika Lackner, Sherezade Moñino-Romero, Ana Maria Giménez-Arnau, Susanne Kimeswenger, Michael Gabriel, Wolfram Hoetzenecker, Sabine Altrichter

**Affiliations:** 1 Department for Dermatology and Venereology, Kepler University Hospital, Linz, Austria; 2 Center for Medical Research (ZMF), Johannes Kepler University, Linz, Austria; 3 Institute of Allergology, Charité—Universitätsmedizin Berlin, Corporate Member of Freie Universität Berlin and Humboldt-Universität zu Berlin, Berlin, Germany; 4 Immunology and Allergology, Fraunhofer Institute for Translational Medicine and Pharmacology ITMP, Berlin, Germany; 5 Department of Dermatology, Hospital del Mar Research Institute, Universitat Pompeu Fabra, Barcelona, Spain; 6 Institute of Nuclear Medicine and Endocrinology, Kepler University Hospital, Linz, Austria; 7 Medical Faculty, Clinical Research Institute for Inflammation Medicine, Johannes Kepler University, Linz, Austria

**Keywords:** basophil activation test, basophils, bee, fcεRI, hymenoptera venom allergy, IgE, wasp

## Abstract

**Background:**

Measurement of IgE levels, skin prick tests (SPT), and basophil activation tests (BAT) are the gold standards for diagnosing Hymenoptera allergy. Currently, BAT most accurately resembles patient reactivity. However, data examining the influence of serum IgE and basophil Fc*ε*RI expression on BAT performance and their clinical relevance are limited.

**Methods:**

Total and specific IgE (sIgE) levels and their ratios, SPT results, high-affinity IgE receptor (Fc*ε*RI) expression on basophils, and BAT results from 31 patients with Hymenoptera-induced anaphylaxis were analyzed and correlated with clinical data.

**Results:**

The sIgE/IgE ratio correlated with positive SPT results at lower venom concentrations in patients with wasp allergy but not in those with bee allergy. Total IgE levels did not correlate with SPT reactivity but correlated with the total (r = 0.742, *p* < 0.01) and IgE-unoccupied Fc*ε*RI (r = –0.796, *p* < 0.01) expression on basophils. The sIgE/IgE ratio significantly correlated with the effector concentration 50 (EC50) at lower wasp venom concentrations and tended to correlate with maximal basophil activation in BAT. A high anaphylaxis grade was associated with low total IgE and high unoccupied Fc*ε*RI levels in patients sensitized to wasp venoms. Furthermore, elevated tryptase levels were associated with low total IgE and Fc*ε*RI levels but high unoccupied Fc*ε*RI levels.

**Conclusions:**

sIgE levels, rather than total IgE levels, can be indicative of functional test results, such as SPT and BAT, in patients allergic to wasp venom but not in those allergic to bee venom. Other activation mechanisms may account for the observed discrepancies in patients allergic to bee venom and should be explored further.

## Graphical Abstract

**Figure:**
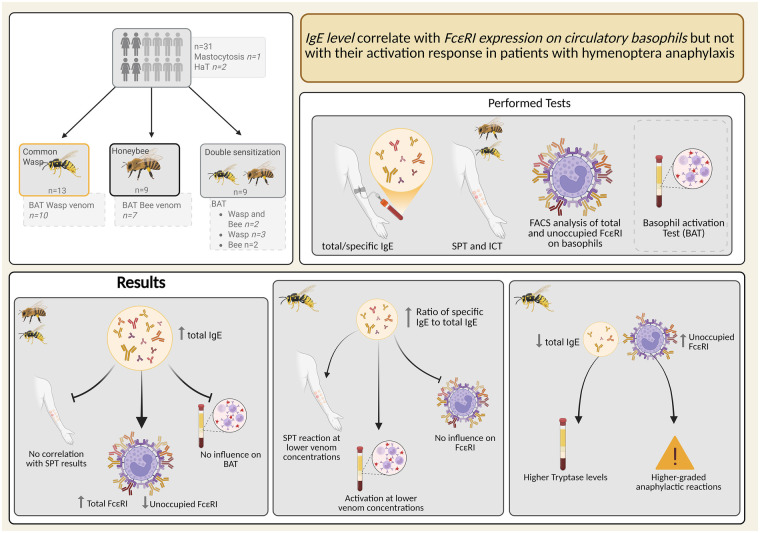

## 1 Introduction

*Hymenoptera* venom allergy (HVA) is a potentially life-threatening condition caused by hypersensitivity to insect stings, such as those of bees, wasps, or hornets. Symptoms vary substantially in severity. Allergic reactions can range from milder symptoms, such as itching or flushing, to life-threatening symptoms, such as swelling of the throat, bronchospasm, hypotonia, and even loss of consciousness (1), which, if left untreated, can eventually result in events such as cardiorespiratory failure (2).

The categorization of anaphylaxis can be difficult and relies on clinical symptoms to grade its severity (3). A grading system according to Ring and Messmer (1) is commonly used because of its simple approach for clinicians with only four categories, although other possibilities for assessing the severity of anaphylaxis have also been described (4).

IgE-mediated mast cell (MC) degranulation and possibly basophil granulocyte activation are considered the main drivers of type I anaphylactic reactions (2). Sensitization to the *Hymenoptera* venom allergen induces the production of specific IgE (sIgE) directed against these antigens. Their binding to the high-affinity IgE receptor Fc epsilon receptor 1 (Fc*ε*RI) on MCs and basophils, followed by the cross-linking of Fc*ε*RI-bound allergen–IgE complexes upon rechallenge, leads to the induction of degranulation of mediators (e.g., histamine), which are the main drivers of the symptoms (2, 5), However, the results of classical allergological tests, such as the determination of total serum IgE and sIgE levels or skin prick tests (SPTs), fail to predict the severity of anaphylactic reactions (6, 7). No data are available demonstrating that the ratio of sIgE to total IgE serves as a better predictor for HVA. For sIgE interpretation, total IgE should be considered to better assess the clinical relevance in individuals with very high or low total IgE levels (8, 9).

MCs are traditionally considered the major effector cells during acute allergic reactions. Furthermore, elevated baseline serum tryptase levels, often caused by genetic mutations, as in mastocytosis or hereditary alpha tryptasemia (HaT), are risk factors for severe anaphylaxis (10). MCs with greater Fc*ε*RI expression, which are more likely to be activated by allergen crosslinking, undergo enhanced degranulation and produce increased cytokine (11, 12). A higher expression of Fc*ε*RI on MCs was observed in patients experiencing wasp venom anaphylaxis than in those without allergy (13). If multiple Fc*ε*RIs are located in proximity, sIgE/Fc*ε*RI cross-linking facilitates clustering, resulting in a high number of sIgE/Fc*ε*RI complexes projected on MCs. This creates a stronger degranulation signal in MCs (14). The presence of IgE is crucial for the increased expression and stabilization of Fc*ε*RI on MCs, thereby preventing Fc*ε*RI degranulation (15).

However, to date, the definitive role of basophils in anaphylaxis remains unclear. Recent studies have indicated a potentially important and specific role for basophils (16). A decrease in circulating basophil numbers *in vivo* in the blood of patients with HVA during anaphylaxis is thought to be a marker for the activation and migration of basophils (17). Fc*ε*RI is expressed at a high level in basophils. Basophil degranulation upon allergen contact is used as a basis for the basophil activation test (BAT) to test for allergic reactions *ex vivo*. In terms of clinical relevance, BAT has better informative value than other standard allergological tests (18). High basophil sensitivity in BAT is a risk factor for severe systemic adverse events during *Hymenoptera* venom immunotherapy (VIT) (19). BAT is particularly useful for the diagnosis of patients with unclear and contradictory histories or sensitization profiles and has been established as the gold standard for such diagnoses. Furthermore, BAT results are associated with VIT efficacy; therefore, they may predict the side effects and treatment failure of VIT (18, 20).

In addition to detecting the insect responsible for the allergic reaction, the presence of IgE is important for Fc*ε*RI expression on basophils (21). It is currently not known whether Fc*ε*RI expression level correlates with BAT test results in patients with insect venom allergy. In this study, we analyzed the correlation between total and sIgE levels induced by insect venom with SPT results, as well as the correlation between total and unoccupied Fc*ε*RI on circulatory basophils in patients with *Hymenoptera* venom anaphylaxis. Furthermore, we analyzed the effects of Fc*ε*RI expression on the standardized BAT test and assessed how the known risk factors for anaphylaxis correlated with all the aforementioned markers.

## 2 Methods

### 2.1 Patients

In this study, we analyzed patients with a history of Hymenoptera venom anaphylaxis who presented to the Comprehensive Allergy Center, Department of Dermatology and Venerology, Kepler University Hospital, Linz, between May 2022 and May 2023. After providing informed consent, 31 patients participated in the study.

Ethical approval for this study was obtained from the local ethics committee (ECS No. 1026/2022). All patient records were handled in a pseudonymized manner, following data protection guidelines and local ethical regulations.

### 2.2 Clinical and laboratory assessments

Patient data, including age, sex, and documented allergies, were obtained from the patient charts. Hymenoptera venom-induced anaphylactic reactions were graded according to the method described by Ring and Messmer (1).

Apart from patients with known mastocytosis or typical cutaneous mastocytosis lesions, all patients without cutaneous mastocytosis lesions but with elevated tryptase levels (>11.4 µg/L) were screened for mastocytosis, which was defined as prevalent if patients presented with a KIT-D816 V mutation (22). In addition, whole blood samples from each patient were screened for HaT using the digital droplet PCR at an external laboratory (MVZ Martinsried GmbH, Martinsried, Germany) (23). HaT was diagnosed if ≥1 additional copy of TPSAB1 was detected.

Total IgE and tryptase serum levels were assessed in the central nuclear laboratory of the Kepler University Hospital, Linz, Austria, using the ImmunoCAP System® (Phadia Laboratory Systems, Thermo Fisher Scientific Inc., Uppsala, Sweden). Furthermore, sIgE levels for wasp and bee extracts [Honeybee, Common wasp (Yellow jacket)] and recombinant allergens (rApi m1, rApi m3, rVes v1, and rVes v5) were analyzed. The levels are expressed in IU/mL.

The ratio of sIgE (extracts and recombinant allergens to total IgE) was calculated for further correlation analysis.

**Figure 1:**
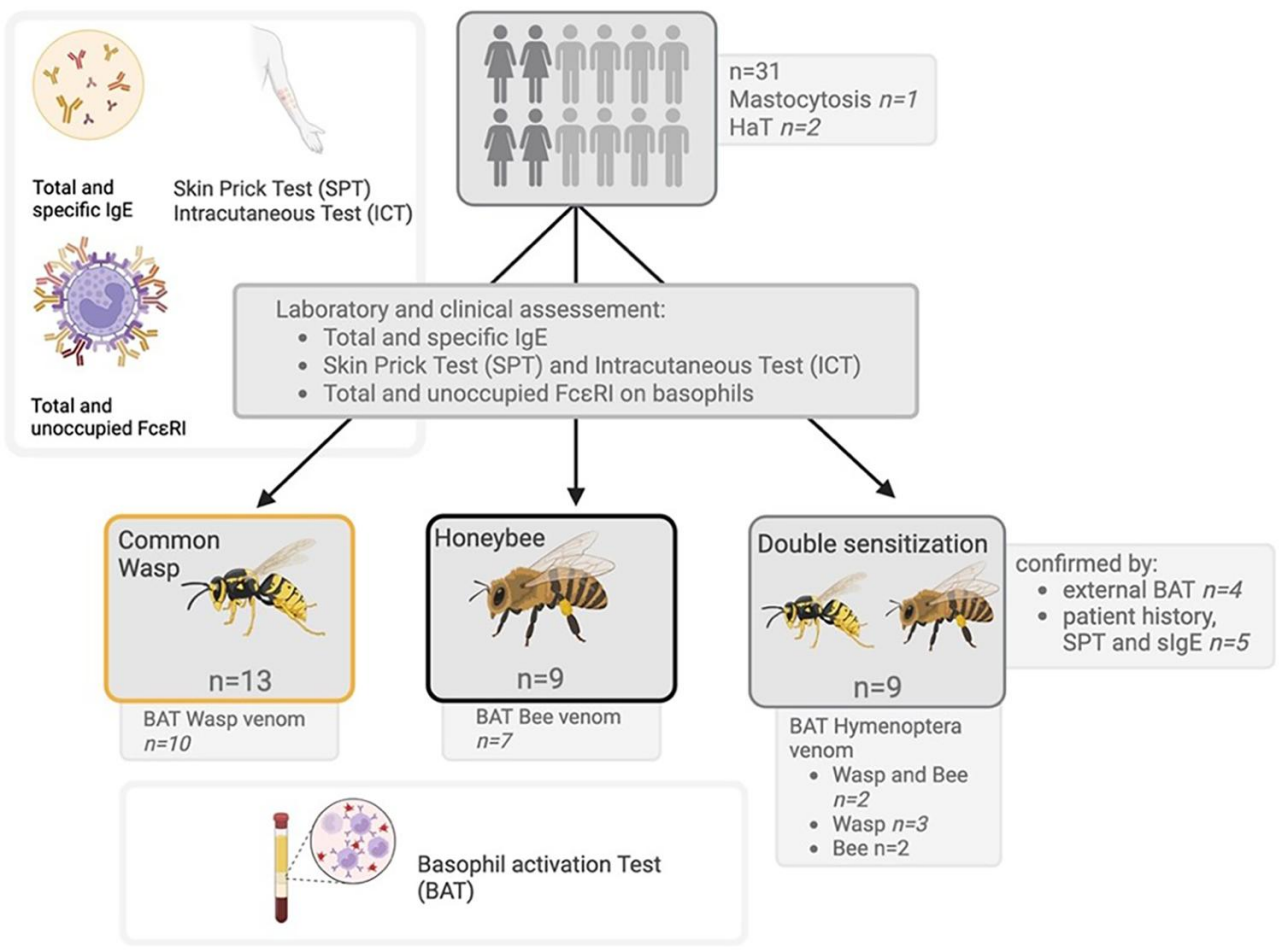
Flowchart of patient grouping. Created in https://BioRender.com. Patients were grouped into wasp venom sensitized (wasp), bee venom sensitized (bee), or both venom/double sensitized (both) according to their medical history, sIgE and recombinant IgE extracts, as well as a positive reaction in the SPT. In cases of inconclusive results, BAT was conducted (Landeskrankenhaus—Universitätsklinik Graz, Department for Dermatology and Venereology) in eight patients. In four of these cases, double sensitization was confirmed via BAT. The additional patients classified as double sensitized had a double positive SPT, sIgE, and a patient history of sting events. BAT, basophil activation test; HaT, hereditary alpha tryptasemia; SPT, skin prick test; sIgE, specific IgE.

The characteristics of the patients are shown in Table 1. Patient grouping was performed as shown in Figure 1.

**Table 1:** Cohort characteristics.

Cohort characteristics	HVA patients (*n* = 31)
Age (y; SD)	48.32 (15.34)
Sex (f, %)	11 (35.5)
BMI (kg/m^2^; SD)	26.86 (3.37) (*n* = 29)
Mastocytosis (pos., %)	1 (3.1)
HaT (pos., %)	2 (6.9)
Other concomitant allergies (pos., %)	11 (39.3)
Total IgE (U/mL; IQR)	56 (130)
Tryptase (µg/L; IQR)	5.1 (3.6)
Hymenoptera sensitization (*n*, %)	
Wasp	13 (42)
Bee	9 (29)
Both	9 (29)
Anaphylaxis (Grade, %)	
I	2 (6.5)
II	24 (77.4)
III	4 (12.9)
IV	1 (3.2)

Results are presented as mean ± SD if normally distributed and median ± IQR if not normally distributed. *n* is given separately if the information was not available for all patients.

HVA, Hymenoptera venom allergy; y, years; SD, standard deviation; f, female; HaT, hereditary alpha tryptasemia; IQR, interquartile range.

### 2.3 SPT and intracutaneous test

SPT (ALK wässrig SQ 801 Bienengift) and intracutaneous test (ICT) were performed with Hymenoptera venoms in 28 patients (ALK wässrig SQ 802 Wespengift) at increasing concentrations. For a detailed protocol, see Supplementary Table 1.

### 2.4 BAT and flow cytometry

BAT was performed using a known sensitizing allergen. In four cases, double-sensitized patients received bee-and-wasp BAT, whereas in the remaining patients, BAT was performed with only one allergen.

A standardized basophil activation test kit (BasoFlowEx Kit; EXIBO) was used with bee (ALK wässrig SQ 801 Bienengift) and wasp allergens (ALK wässrig SQ 802 Wespengift) at a concentration of 100 µg/mL each to conduct BAT. Bee and wasp venoms were diluted with ALK diluent to generate concentrations of 100, 10, 1, 0.1, and 0.01 µg/mL. Patient blood was drawn into heparinized collection tubes, serially diluted with venom, and processed according to the manufacturer’s instructions. Subsequently, the probes were analyzed using flow cytometry (Beckman Coulter DxFlex). The gating strategy is illustrated in Supplementary Figure 1a. Monoclonal anti-IgE antibodies from the Basophil Test Kit were used as positive controls.

Positive and negative controls were used for each case. Seven individuals were excluded from further statistical analysis due to high preactivation or a lack of basophil response during BAT (Supplementary Figure 2).

Excel was used to calculate the dilution interval [<0.01, 0.1–0.01, 1–0.1, 10–1, 100–10, 100 µg/mL (undiluted)] for BAT EC50, allergen concentration of maximal, and EC50 of basophil activation, based on FACS data.

The concentration at which maximum BAT activation was first achieved was determined using a 10% tolerance range (Supplementary Figure 2).

### 2.5 Fc*ε*RI expression profile measurements

To evaluate the Fc*ε*RI density on basophil granulocytes, whole blood samples were collected from patients in heparin-containing tubes. Human immunoglobulin (IG VENA 50 g/L; Kedrion, Bologna, Italy) was used as the blocking agent. For FACS, anti-human CD193-APC (5E8, Cat. No. 558208; BD) and anti-human CD123-PE (9F5; Cat. No. 555644, BD) were used. CRA1-BV421 antibody (Cat. No. 334624; BioLegend) was used for total Fc*ε*RI staining, while the CRA2-FITC antibody (Cat. No. GTX00853; GeneTex) was used for unoccupied Fc*ε*RI staining. The isotype control antibodies used were IgG2b-BV421 (MPC-11, Cat. No. 400307; BioLegend) and IgG1-FITC (IC002F, R&D Systems). The MFIs of CRA1 (total Fc*ε*RI) and CRA2 (unoccupied Fc*ε*RI) were assessed using FACS, and the gating strategy is illustrated in Supplementary Figure 1b.

Quantification beads (Quantum Simply Cellular anti-Mouse IgG, Cat. No. 815B; Bangs Laboratories, Inc.) with defined numbers of Fc*ε*RI on their surface were used together with the calculation tool QuickCal v 2.3 to transform MFI into the unit “receptors per basophil granulocyte.” Quantification was conducted seven times in parallel with FACS analysis of patient samples using CRA1 and CRA2 antibodies over approximately 1 year. The mean of the measurements was used to transform the MFI into the number of receptors per cell. Five patients showed slightly negative MFI on FACS, hindering the transformation of the data into the number of receptors per cell; therefore, the number of receptors per cell was defined as zero.

### 2.6 Statistical analyses

Statistical analyses were performed using ISM SPSS Statistics version 30.0.0.0. Normal distribution was determined using the Kolmogorov–Smirnov test. Normally distributed variables are reported as median ± standard deviation (SD), while non-normally distributed variables are reported as median and interquartile range (IQR). Correlations were calculated using Spearman’s rho test. Binomial variables were analyzed using the Mann–Whitney U test. A *p*-value ≤ 0.05 was considered statistically significant.

## 3 Results

### 3.1 Broad range of total and sIgE levels in patients with Hymenoptera venom allergy

In patients with HVA, total IgE levels were elevated (mean  ± SD, 144 IU/mL ± 240). However, a 100-fold difference between the lowest (11 IU/mL) and highest total IgE levels (1087 IU/mL) was observed in these patient samples, without any differences between the HVA groups (Supplementary Figure 3a). The sIgE levels and the ratio of sIgE to total IgE were significantly higher in the HVA group. On average, double-sensitized patients had lower sIgE levels for both insect venoms than monosensitized patients (Supplementary Figures 3a,b).

### 3.2 Significant correlation between the ratio of sIgE/total IgE and SPT results in patients with wasp anaphylaxis

Total IgE and sIgE levels in the respective allergen extracts (honeybee or common wasp) or recombinant allergens (rApi m1 and rApi m3 for bee allergens; rVes v1 and rVes v5 for wasp allergens) did not correlate with SPT results. Only the ratio of sIgE/total-IgE for the wasp venom extract significantly correlated with the SPT findings (Table 2). A higher ratio correlated with a positive SPT at higher venom dilutions (i.e., higher cutaneous sensitivity to the allergen extract; Supplementary Figure 3c). Such a correlation was not observed for the bee venom (Supplementary Figure 3d).

**Table 2:** Correlation of total IgE and sIgE/total IgE ratios with skin prick/ICT results.

Variable	Wasp venom-sensitized patients SPT/ICT wasp (*n* = 21^a^)
Total IgE	−0.018
sIgE wasp extract	−0.308
Wasp sIgE to total IgE ratio	**−0** **.** 503*
sIgE rVes v1	−0.002
sIgE rVes v 1 to total IgE ratio	−0.004
sIgE rVes v5	−0.253
sIgE rVes v5 to total IgE ratio	−0.378

	Bee venom-sensitized patients SPT/ICT bee (*n* = 18)
Total IgE	−0.0.07
sIgE bee extract	−0.078
sIgE bee to total IgE ratio	0.070
sIgE rApi m1	−0.258
sIgE rApi m1 to total IgE ratio	−0.304
sIgE rApi m3	−0.208
sIgE rApi m3 to total IgE ratio	−0.087

*R*-values of Spearman’s rho correlation (two-sided) are presented. *p < 0.05.

Values in bold indicate statistical significance.

a One patient did not receive SPT.

SPT, skin prick test; ICT, intracutaneous test.

### 3.3 Strong impact of total IgE serum levels on Fc*ε*RI expression of circulating basophils

**Figure 2:**
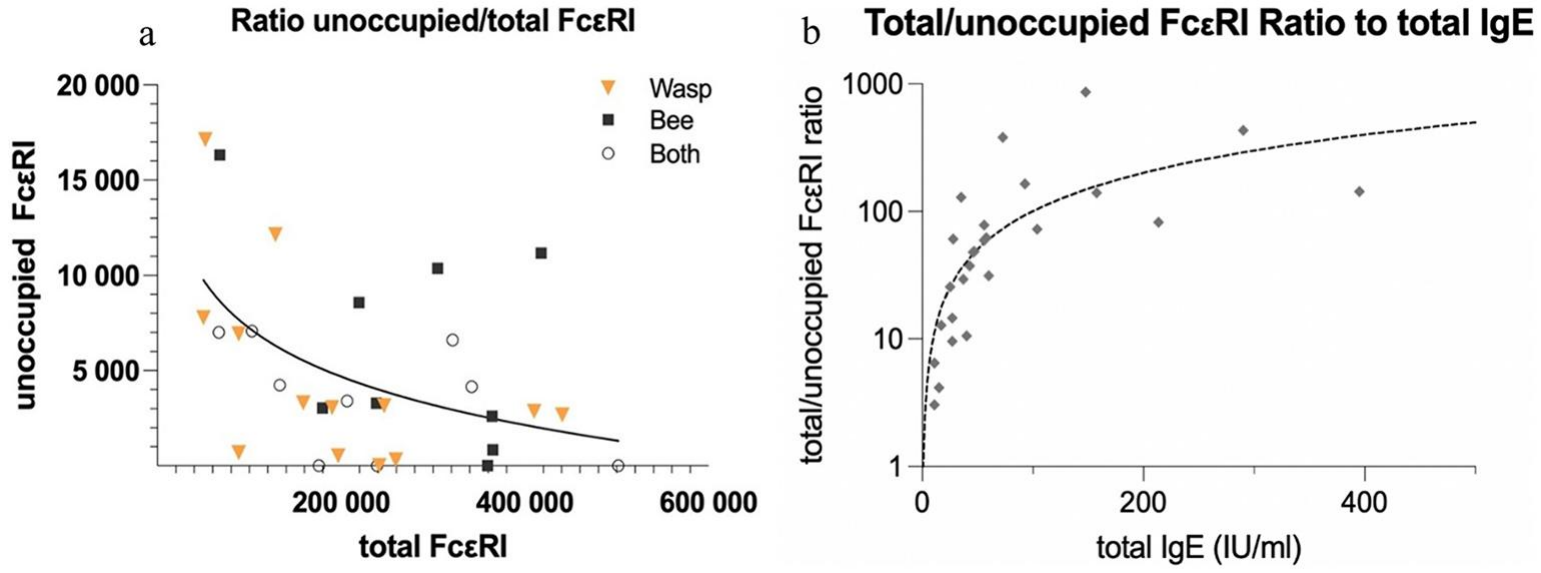
Fc*ε*RI correlation. **(a)** Correlation of unoccupied and total Fc*ε*RI levels. Each person is displayed as an individual symbol with specific Hymenoptera sensitization. The black line shows a non-linear regression curve (correlation coefficient −0.441; *p* < 0.05). **(b)** Unoccupied/total Fc*ε*RI ratio depicted on a logarithmic scale correlated to total IgE (IU/mL). The line of identity is shown. Fc*ε*RI is shown as a receptor per basophil.

Basophils in patients with HVA exhibited a broad range of total Fc*ε*RI expression levels. The range was approximately 50,000 to more than 500,000 receptors/cell (Supplementary Figure 4A). In most patients, most Fc*ε*RIs were occupied with IgE, and the median of the free unoccupied Fc*ε*RI was 1.6% (IQR 3.65) (Supplementary Figure 4). No significant differences were observed between the HVA groups (Supplementary Figure 4B). A high total Fc*ε*RI level was significantly and inversely correlated with a low unoccupied Fc*ε*RI level (Figure 2a).

Total IgE serum levels were strongly and significantly correlated with the total (*r* = 0.742, *p* < 0.01) and unoccupied (*r* = –0.796, *p* < 0.01) Fc*ε*RI expression and their respective ratios (*r* = 0.857, *p* < 0.01) on basophils (Figure 2b), irrespective of the HVA group (Table 3).

**Table 3:** Correlation of Fc*ε*RI density with total and specific IgE to Hymenoptera venom extracts.

Wasp venom-sensitized patients	Total FcεRI (*n* = 22)	Unoccupied FcεRI (*n* = 22)	Total/unoccupied FcεRI ratio (*n* = 18^a^)
		Correlation coefficient	
Total IgE	0.834**	−0.776**	0.825**
Wasp sIgE	0.440*	−0.448*	0.463
Wasp sIgE/ total IgE ratio	−0.173	−0.031	−0.036

Bee venom-sensitized patients	Total FcεRI (*n* = 18)	Unoccupied FcεRI (*n* = 18)	Total/unoccupied FcεRI ratio (*n* = 14^a^)
Total IgE	0.662**	−0.840**	0.889**
Bee sIgE	0.565*	−0.354	0.515
Bee sIgE/ total IgE ratio	0.051	0.275	−0.314

*R*-values of Spearman’s rho correlation (two-sided) are presented. *p < 0.05 and **p < 0.01.

Values in bold indicate statistical significance.

a As five patients did not have detectable levels of unoccupied FcεRI, the ratio could not be calculated.

### 3.4 Association between higher sIgE levels to bee and wasp allergens and total Fc*ε*RI expression on basophils

Serum sIgE levels against bee and wasp venom were significantly correlated with the total Fc*ε*RI expression on basophils (Table 3). Their respective ratios with total IgE did not correlate, indicating that their contribution to the total IgE level influences Fc*ε*RI receptor density without eliciting other specific effects. A positive correlation was observed between recombinant sIgE rVes v5 and rApi m1 with total Fc*ε*RI, but not between the recombinant sIgE rVes v5 and rApi m1 and the total IgE ratio with total Fc*ε*RI. Furthermore, the rApi m3 levels were inversely correlated with the amount of unoccupied Fc*ε*RI levels (Supplementary Table 2 and Supplementary Figure 5).

### 3.5 Influence of the sIgE (wasp venom extract) to total IgE ratio on BAT test results

**Figure 3:**
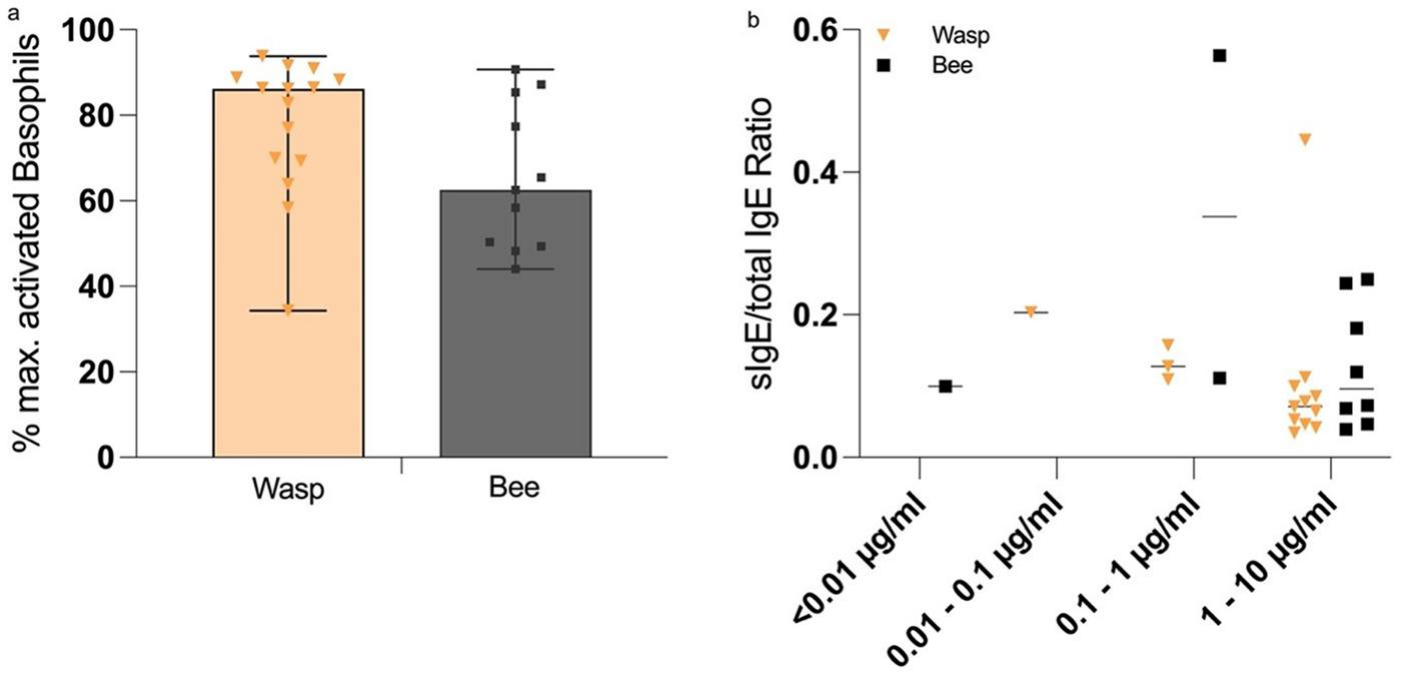
Basophil activation test (BAT). **(a)** Percentage of maximum activated basophils at any concentration. **(b)** Ratio of sIgE (wasp and bee extracts)/total IgE at the concentration range/step of EC50.

Interpretable BAT test results were obtained in 15 of 22 patients with wasp allergy and in 11 of 18 patients with bee allergy. Patients in both HVA groups showed high maximal basophil activation (Figure 3a). Maximal basophil activation did not significantly correlate with total IgE, sIgE, SPT, or Fc*ε*RI expression. The ratio of sIgE (to wasp venom extract) to total IgE correlated with the maximal percentage of activated basophils (Supplementary Table 3a).

In patients allergic to wasp venom, the venom dilution step at which the EC50 of the basophils occurred (EC50 venom concentration range) was significantly inversely correlated with the sIgE/total IgE levels (*r* = 0.604, *p* = 0.017) (Figure 3b and Supplementary Table 3b). Similarly, the ratio of rVes v5 to total IgE was inversely correlated with the venom concentration (Supplementary Table 3b). A similar trend was observed using the calculated venom concentrations (Supplementary Table 3b and Supplementary Figure 5).

In the bee-sensitized cohort, a significant correlation was only observed between the total/unoccupied Fc*ε*RI ratio and venom concentration (*p* = 0.024) (Supplementary Table 3b). No other correlations were observed in the BAT results of the 11 patients with bee allergy.

No correlation was observed between the EC50 concentration and total IgE Fc*ε*RI expression on basophils or the clinical data of the patients (Supplementary Figure 6).

### 3.6 Influence of known risk factors on the IgE, Fc*ε*RI, and BAT results

Male sex is a known risk factor for HVA. This was reflected in our patient cohort, which included significantly more male than female patients sensitized to bee venom. However, sex did not affect the parameters measured in this study (Table 4).

**Table 4:** Influence of known risk factors on the IgE, FcεRI, and BAT results.

Wasp-sensitized patients (*n* = 22)	Sex	Age	Tryptase	Anaphylaxis grade
	*p*-Value (MWU)	Correlation coefficient		
Age	0.59	-	0.220 (0.33)	0.390 (**0**.**07**)
Tryptase	0.43	0.220 (0.33)	-	0.659** (<0.0.1)
Anaphylaxis grade	0.22	0.390 (0.**07**)	0.659** (<0.0.1)	**-**
Total IgE	0.29	−0.318	−0.457* (0.03)	−0.535* (0.01)
sIgE wasp	0.06	0.048	−0.167	−0.231
sIgE ratio wasp/total IgE	0.22	0.472* (0.03)	−0.027	0.041
Total FcεRI	0.838	−0.364	−0.443* (0.4)	−0.342
Unoccupied FcεRI	0.355	0.075	0.468* (0.03)	0.452* (0.04)
Total/unoccupied FcεRI ratio	0.497^a^	−0.175^a^	−0.571^a^* (0.01)	−0.446^a^
EC50 BAT	0.713^#^	−0.284^#^	0.129^#^	0.035^#^
Max. activation BAT	0.540^#^	−0.179^#^	−0.065^#^	−0.070^#^

Bee-sensitized patients (*n* = 18)	Sex	Age	Tryptase	Anaphylaxis grade
Age	**0**.04*	-	0.535* (0.02)	0.318 (0.2)
Tryptase	0.55	0.535* (0.02)	-	**0**.652** (0.01)
Anaphylaxis grade	0.76	0.318 (0.2)	0.652** (0.01)	**-**
Total IgE	0.59	0.069	−0.047	−0.349
sIgE bee	0.69	−0.145	−0.021	−0.419
sIgE bee/total IgE ratio	0.81	−0.254	−0.244	−0.400
Total FcεRI	0.35	0.248	−0.085	−0.318
Unoccupied FcεRI	0.62	−0.154	0.030	0.043
Total/unoccupied FcεRI ratio	0.78^a^	0.227^a^	−0.084^a^	−0.254^a^
EC50 BAT	0.85^b^	0.155^b^	−0.018^b^	0.273^b^
Max activation BAT	0.45^b^	−0.036^b^	−0.269^b^	−0.319^b^

*p*-Values (two-sided) of the Mann–Whitney-U test (MWU) for binomial variables (sex) and *R*-values of Spearman’s rho correlation (two-sided) are presented. *p < 0.05 and **p < 0.01.

EC50, effector concentration 50 (half-maximal activation in BAT).

Values in bold indicate statistical significance.

a As five patients did not have detectable levels of unoccupied FcεRI, the ratio could not be calculated.

b Excluding patients due to high preactivation or lack of basophil response during BAT.

Advanced age is associated with an elevated grade of anaphylaxis. However, no such correlations were observed in the present cohort. Older individuals in the wasp allergy patient group had significantly higher sIgE to total IgE ratios and tryptase levels. In patients with bee allergy, age was also positively correlated with tryptase levels; however, it did not affect any of the parameters assessed in this study.

Elevated tryptase levels are also a risk factor for severe anaphylaxis, as observed in this cohort. In patients with wasp allergy, the total IgE level was significantly lower than that in patients with a higher tryptase level. Furthermore, a positive correlation was observed between tryptase levels and unoccupied Fc*ε*RI levels. This correlation was not observed in patients with bee allergy.

Patients with wasp allergy and a high anaphylaxis grade had significantly lower total IgE levels and a high number of unoccupied Fc*ε*RI on basophils. In patients with bee allergy, no clear correlation was observed between the aforementioned risk factors and total or specific IgE levels. In this cohort, no correlation was observed between BAT results and any of the assessed parameters (Table 4).

## 4 Discussion

In this study, we examined a typical local cohort of patients with HVA. In the HVA cohort, approximately two-thirds were male, who more frequently exhibited wasp venom sensitization. The patients exhibited a broad range of total and sIgE levels against venom extracts and recombinant allergens. Genetic predisposition and coexisting allergic conditions seem to have a significant influence (24). Although sIgE levels are helpful for diagnosing insect allergy (25), functional assays such as SPT (26) and BAT (18) are superior for diagnosing relevant allergies. In our cohort, the ratio of sIgE to total IgE, but not the sole sIgE levels, significantly correlated with SPT results in patients with wasp venom allergy. However, this correlation was not observed in patients with a bee venom allergy. By and large, bee and wasp venom allergy share common IgE-mediated pathomechanisms (27) but also have well-known clinical differences, such as more side effects and reduced response rates of specific immunotherapy in bee-allergic patients (28). Advanced diagnostics have shown that true double sensitization to both venoms is rare, despite the fact that the patients have often been stung by both insects (29). Some evidence exists that bee and wasp venom contains possibly costimulatory substances (30–33) that may lead to distinct molecular sensitization profiles and effector cell activation pathways (34). The differences observed in our study between wasp and bee allergic patients also point toward distinct mechanisms of bee and wasp allergy. At the same time, the small cohort size and heterogeneity remain potential biases.

Total IgE levels significantly correlated with total and unoccupied Fc*ε*RI expression on circulating basophils in patients with HVAs. Similar correlations have been reported in patients with chronic urticaria (35), allergic rhinitis (36), or atopy (37, 38) but not in patients with HVA. A higher number of IgE stabilizes Fc*ε*RI, thereby preventing its degradation (14). sIgE levels were elevated in response to bee and wasp allergen extracts, but their proportion relative to total IgE was not elevated; however, sIgE levels were associated with total Fc*ε*RI expression. These findings suggest that allergen-specific IgE, in addition to contributing to the overall IgE pool, may influence the receptor expression. However, no other evidence for an additional effect on Fc*ε*RI expression was identified.

Prussin et al. (39) found a correlation between Fc*ε*RI expression and increased basophil activation. However, in this study, we did not find a correlation between the SPT results, or Fc*ε*RI expression, and BAT results. We observed a trend toward significance between the BAT response and the ratio of specific IgE to total IgE in patients sensitized to wasp venom. In contrast, total IgE or specific IgE levels alone did not significantly correlate with BAT results. It is possible that the ratio of sIgE to total IgE could be helpful in better assessing the clinical significance of sensitization. This may be relevant for patients with very high or very low total IgE levels. Very high total IgE values, as found in patients with atopic dermatitis, are often associated with multiple sensitizations of questionable clinical relevance (8), whereas in patients with very low total IgE, low sIgE levels could underestimate relevant sensitizations (9).

Basophil activation following allergen exposure depends on multiple factors, including the proportion of Fc*ε*RI occupied by sIgE, affinity and clonality of IgE antibodies, receptor cross-linking efficiency, intracellular signaling, and the activation threshold of individual basophils, rather than being determined by Fc*ε*RI expression levels alone (40–43). Consequently, increased receptor expression may enhance the potential for activation without necessarily translating directly into functional responses measured by the BAT. Indeed, our findings indicate that Fc*ε*RI expression primarily correlates with IgE levels but not with cell activation. BAT may capture a broader picture of the functional consequences of multiple regulatory processes acting downstream of the receptor. Overall, significant correlations between sIgE levels and functional test results (SPT and BAT) were observed only in patients with wasp venom allergies. A similar positive association between sIgE and basophil activation (CD63+) has been reported in wasp allergy (44). In addition, the ratio of sIgE to total IgE concentrations appeared to be significantly related to the venom concentration required to reach EC50 in BAT. However, sIgE values alone are not considered reliable predictors of anaphylactic severity upon re-exposure to the source antigen (45). Activation of alternative surface receptors during anaphylaxis, such as the Mas-related G protein-coupled receptor X2 (46), independent of IgE, may contribute to the absence of correlation between IgE levels and clinical severity. Bee venom has also been proposed to directly induce MC activation through a non-IgE-mediated mechanism (47), which could partly explain why associations between sIgE levels and the results of functional assays are not evident in bee venom allergies.

BAT measures the responsiveness of circulating basophils following *in vitro* allergen stimulation, whereas SPT primarily reflects the activation of tissue-resident skin mast cells *in vivo*. Furthermore, the emergence of mast cell activation tests (MAT) has reinforced the concept that basophils and mast cells can exhibit differential activation patterns even in the same allergic individual, suggesting that these cell types may contribute complementary rather than identical information regarding effector cell function (48, 49).

However, because our study did not directly assess mast cell function but primarily assessed basophil responses, the present data cannot distinguish whether the observed BAT and SPT discrepancies arise predominantly from the intrinsic biological differences between basophils and mast cells or from methodological differences between the assays. Future studies incorporating parallel BAT and MAT, together with SPT analyses, may help clarify the relative contributions of these mechanisms.

Male sex, a known risk factor for anaphylaxis (50), did not influence any of the assessed parameters. However, elevated tryptase levels were strongly correlated with a higher anaphylaxis grade, as previously described (50). Furthermore, tryptase values and anaphylaxis grade correlated with the ratio of unoccupied/total Fc*ε*RI in patients with wasp allergy. We also noted a trend toward a correlation between high numbers of unoccupied Fc*ε*RI and high anaphylaxis grades, although we did not observe any association between BAT results and severity of anaphylaxis. This finding is consistent with those of previous reports, which described BAT as a useful tool to identify sensitization to Hymenoptera, but with no correlation to the symptoms during anaphylaxis (51, 52). In contrast, a connection between peanut anaphylaxis severity and the threshold of allergic reactions in children, as well as the percentage of CD63+-activated basophils, has been established previously (53).

The small number of patients recruited is a major limitation of this study. Furthermore, we did not have a control group, as we did not aim to identify patients with HVA but aimed to better characterize these patients and the correlation between different test results. Approximately 84% of patients experienced low-grade anaphylaxis (Grades 1 and 2), and severe anaphylaxis was underrepresented. Wasp venom allergy was not assessed or discriminated for *Polistes* sensitization. One factor that can be examined in future studies is the cause of the very high venom concentration required to reach EC50 in this patient cohort (Figure 3). Nonetheless, this study provides a rationale for future research in this area. Future studies should include a larger number of patients and controls. The study did not assess the risk factors for missing skin symptoms or the time point of the last stinging reaction, as most patients could not recall the events in detail. A strength of this study was the in-house assessment of all assays with the same allergen source, providing short transportation of basophils and timely analysis within a few hours after blood collection.

Taken together, this study showed that total and specific IgE levels affect Fc*ε*RI expression on basophils, as well as SPT and BAT results. However, a direct influence of Fc*ε*RI expression on BAT results was not observed. The proportion of specific IgE to total IgE was superior to that of specific IgE alone and correlated with the functional assay only in patients with wasp allergy. For patients with bee allergies, additional non-IgE-mediated mechanisms may be important, which requires further exploration.

## Data Availability

The raw data supporting the conclusions of this article will be made available by the authors, without undue reservation.
