# Supplementary material for: IgE levels correlate with FcεRI expression on circulatory basophils but not with their activation response in patients with Hymenoptera anaphylaxis
Source: Front Allergy. 2026 Jul 20;7:1864564. doi: 10.3389/falgy.2026.1864564 (PMC13430313; doi:10.3389/falgy.2026.1864564)
Supplement: Supplementary file 1 [file Supplementaryfile1.docx]

**Supplements – Frontiers in Allergy**

**IgE level correlate with FcεRI expression on circulatory basophils but not with their activation response in patients with hymenoptera anaphylaxis**

Stefan Aigner ^#^ ^1,2^, Viktoria Puxkandl ^#^ ^1,2^, Teresa Burner^1,2^, Angelika Lackner^1,2^, Sherezade Moñino-Romero^3,4^, Ana Maria Giménez-Arnau^5^, Susanne Kimeswenger^1,2^, Michael Gabriel^6^, Wolfram Hoetzenecker^1,2,7^_,_ Sabine Altrichter^1,2,3,4,7^

1. Department for Dermatology and Venereology, Kepler University Hospital, Linz, Austria
2. Center for medical research (ZMF), Johannes Kepler University, Linz, Austria
3. Institute of Allergology, Charité - Universitätsmedizin Berlin, Corporate Member of Freie Universität Berlin and Humboldt-Universität zu Berlin, Berlin, GermanyInstitute of Allergology, Charité – Univer-sitätsmedizin Berlin, corporate member of Freie Universität Berlin, Humboldt -Universität zu Berlin, and Berlin Institute of Health, Berlin, Germany
4. Fraunhofer Institute for Translational Medicine and Pharmacology ITMP, Immunology and Allergology IAAllergology and Immunology, Berlin, Germany
5. Department of Dermatology, Hospital del Mar Research Institute, Universitat Pompeu Fabra, Barcelona
6. Institute of Nuclear Medicine and Endocrinology, Kepler University Hospital, Linz, Austria
7. Clinical Research Institute for Inflammation Medicine, Medical Faculty, Johannes Kepler University, Linz, Austria

# Contributed equally (Co-Authorship)

***Repository Tables***

|  | Venom | Dosage | Application |
| --- | --- | --- | --- |
| 1 | **Bee venom** | 1µg | SPT |
| 2 | **Bee venom** | 10µg | SPT |
| 3 | **Bee venom** | 100µg | SPT |
| 4 | **Bee venom** | 300µg | SPT |
| 5 | **Bee venom** | 0,1µg | ICT |
| 6 | **Bee venom** | 1µg | ICT |
| 1 | **Wasp venom** | 1µg | SPT |
| 2 | **Wasp venom** | 10µg | SPT |
| 3 | **Wasp venom** | 100µg | SPT |
| 4 | **Wasp venom** | 300µg | SPT |
| 5 | **Wasp venom** | 0,1µg | ICT |
| 6 | **Wasp venom** | 1µg | ICT |
|  | **Positive** control |  | SPT |
|  | **Negative** control | 10mg/ml | SPT |

**Repository Table 1** - Skin Prick-Test (SPT) and Intracutaneous-Test (ICT) scheme using ALK wässrig® SQ bee and wasp venom.
As positive control served histamine (0.1%) and as negative control saline solution (sodiumchloride 0.9%) from Fresenius SE & Co. KGaA, Bad Homburg, Germany. Wheal development 1.5 mm bigger than the saline prick after 20 minutes was considered positive. Tests were stopped when patients developed wheals bigger than 4 mm (SPT) or 8 mm (ICT). The dilution step in which the reaction was first clearly positive (wheal size ≥3mm) was used for further correlation analysis.

| Wasp venom sensitized Patients | *Total FcεRI*  (n=22) | *Unoccupied FcεRI*  (n=22) | *Unoccupied/total FcεRI ratio* (n=18^a^) |
| --- | --- | --- | --- |
|  | Correlation Coefficient | | |
| sIgE rVes v1 | .196 | -.306 | .322 |
| sIgE rVes v1/ total IgE ratio | -.365 | .212 | -.104 |
| sIgE rVes v5 | **.515*** | -.407 | .429 |
| sIgE rVes v5/ total IgE ratio | -.027 | .023 | -.044 |
|  |  |  |  |
| Bee venom sensitized Patients | *Total FcεRI*  (n=18) | *Unoccupied FcεRI*  (n=18) | *Unoccupied/total FcεRI* ratio (n=14^a^) |
| sIgE rApi m1 | **.597**** | -.391 | .389 |
| sIgE rApi m1/total IgE ratio | -.320 | -.038 | -.009 |
| sIgE rApi m3 | .376 | **-.576*** | .507 |
| sIgE rApi m3/ total IgE ratio | -.112 | -.030 | -.147 |

**Repository Table 2** – Correlation of FcεRI expression with sIgE to recominant allergens

R-Values of the Spearman-Rho correlation (2-sided) are given. p-Values are given as * <0.05, **<0.01.

^a^ Since 5 patients did not have a detectable level of unoccupied FcεRI, the ratio could not be calculated.

| Wasp venom sensitized Patients (n=15) | Max. activated basophils (%) | p-value |
| --- | --- | --- |
| Correlation Coefficient | | |
| Total IgE | .011 | .97 |
| sIgE Wasp | .300 | .28 |
| ratio sIgE Wasp/total IgE | **.504** | **.06** |
| sIgE rVes v 1 | .176 | .53 |
| sIgE rVes v 1/ total IgE ratio | .189 | .50 |
| sIgE rVes v5 | .154 | .59 |
| sIgE rVes v5/ total IgE ratio | .250 | .37 |
| Prick-Test Wasp | -.255 | .38 |
| Total FcεRI | -.268 | .33 |
| Unoccupied FcεRI | -.181 | .52 |
| Total/unoccupied FcεRI ratio | -.016^a^ | .96 |
|  |  |  |
| Bee venom sensitized Patients (n=11) | Max. activated basophils (%) | p-value |
| Total IgE | -.082 | .81 |
| sIgE Bee | .127 | .71 |
| ratio sIgE Bee/total IgE | .245 | .47 |
| sIgE rApi m1 | .059 | .86 |
| sIgE rApi m1/total IgE ratio | -.096 | .78 |
| sIgE rApi m3 | -.124 | .72 |
| sIgE rApi m3/total IgE ratio | -.136 | .69 |
| Prick-Test Bee | .118 | .73 |
| Total FcεRI | .309 | .36 |
| Unoccupied FcεRI | .218 | .52 |
| Total/unoccupied FcεRI ratio | .139^a^ | .70 |

**Repository Table 3a**- Correlation of maximum activated basophils (%) at any concentration in basophil activation test (BAT) with total IgE, sIgE recombinant allergens and FcεRI expression.
R-Values of the Spearman-Rho correlation (2-sided) are given. p-Values are given as * <0.05, **<0.01.
^a^ Since 5 patients did not have a detectable level of unoccupied FcεRI, the ratio could not be calculated.

| Wasp venom sensitized Patients (n=15) | | EC50 (concentration step/range^b^) | p-value | EC50 (calculated concentration) | p-value |
| --- | --- | --- | --- | --- | --- |
|  | **Correlation Coefficient** | | | | |
| Total IgE | | -.090 | .75 | .164 | .56 |
| sIgE Wasp | | .327 | .23 | -.039 | .89 |
| ratio sIgE Wasp/total IgE | | **.604*** | .**02** | -.507 | **.05** |
| sIgE rVes v 1 | | -.170 | .55 | .270 | .33 |
| sIgE rVes v 1/ total IgE ratio | | -.129 | .65 | .075 | .79 |
| sIgE rVes v5 | | .360 | .19 | .018 | .95 |
| sIgE rVes v5/ total IgE ratio | | **.604*** | **.02** | -.325 | .24 |
| Prick-Test Wasp | | -.261 | .37 | .501 | **.07** |
| Total FcεRI | | -.083 | .77 | .264 | .34 |
| Unoccupied FcεRI | | -.187 | .51 | -.055 | .85 |
| Total/unoccupied FcεRI ratio | | .00^a^ | 1.0 | .099^a^ | .75 |
|  | |  |  |  |  |
| Bee venom sensitized Patients (n=11) | | EC50 (concentration step/range^b^) | p-value | EC50 (calculated concentration) | p-value |
| Total IgE | | -.319 | .34 | .164 | .63 |
| sIgE Bee | | -.029 | .93 | -.273 | .42 |
| ratio sIgE Bee/total IgE | | .215 | .53 | -.427 | .19 |
| sIgE rApi m1 | | .145 | .67 | -.465 | .15 |
| sIgE rApi m1/total IgE ratio | | .314 | .35 | .064 | .85 |
| sIgE rApi m3 | | .018 | .96 | -.221 | .51 |
| sIgE rApi m3/total IgE ratio | | .447 | .17 | -.527 | **.10** |
| Prick-Test Bee | | -.313 | .35 | .207 | .54 |
| Total FcεRI | | -.203 | .55 | .027 | .94 |
| Unoccupied FcεRI | | .261 | .44 | -.227 | .50 |
| Total/unuoccupied FcεRI ratio | | **-.701*** | **.02** | .564^a^ | **.09** |

**Repository Table 3b** - Correlation of half-max. (EC50) basophil activation (concentration step/range^b^; calculated concentration) in BAT with total IgE, sIgE recombinant allergens and FcεRI expression.
 R-Values of the Spearman-Rho correlation (2-sided) are given. p-Values are given as * <0.05, **<0.01.
EC50 – effective concentration 50 (half-max basophil activation)
^a^ Since 5 patients did not have a detectable level of unoccupied FcεRI, the ratio could not be calculated.

^b^ Concentration steps: <0.01 µg/ml, 0.1-0.01 µg/ml, 1-0.1µg/ml, 10-1 µg/ml, 100-10 µg/m.

***Repository Figures***


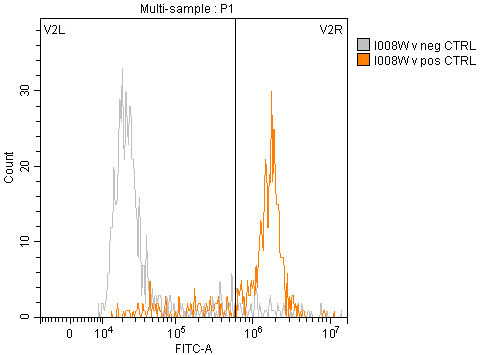

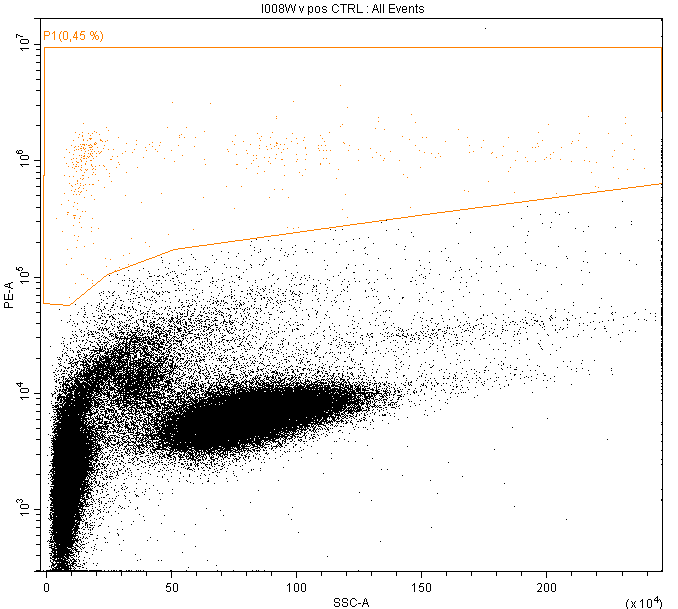


**Repository Figure 1a** - *Gating strategy basophil activation test.*
A standardized basophil activation test kit from EXIBO© (BasoFlowEx Kit Ref. ED7043) was used (bee and wasp allergen from the company ALK (ALK wässerig SQ© 801 Bienengift 100µg/ml & ALK wässerig SQ© 802 Wespengift 100µg/ml)). After stimulation according to the kit the probes were analyzed by flow cytometry (Beckman coulter DxFlex©).

For analyzing the FACS data, the application CytExpert© was chosen. The used Gain settings are stated here: FSC, SSC, CD63 (FITC) , CD203c (PE). The Compensation Matrix was not altered (= 0,00 for all values of the Compensation Matrix).

b) The read out of the basophil activation test is the percentage of CD63 positive cells (orange curve). The gating was set at the 5th percentile of the negative control (grey curve), below which 95% of the unstimulated basophils fall.

a

b


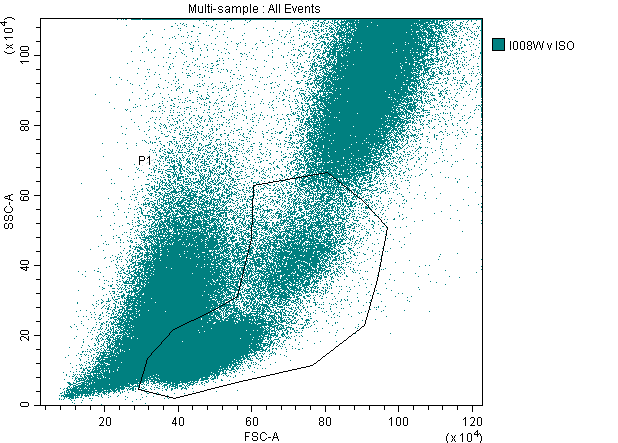

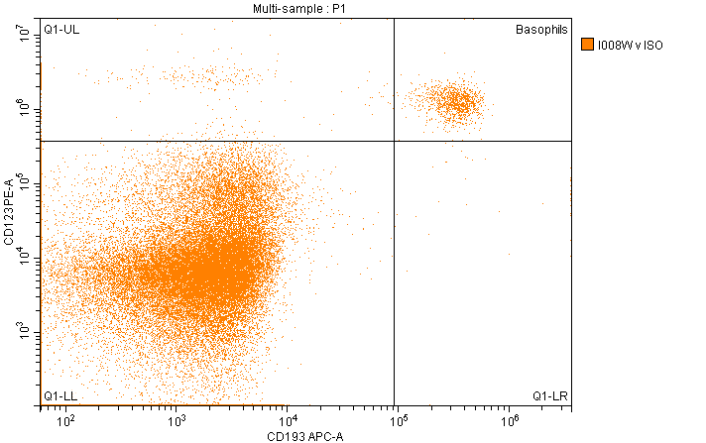


a

b

c


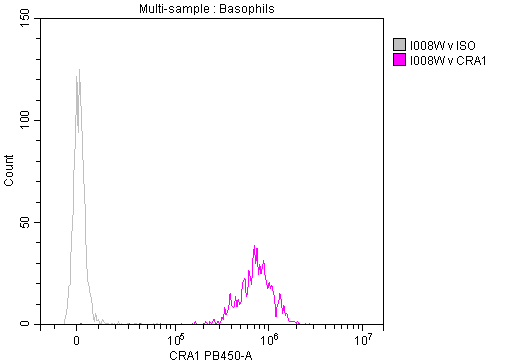

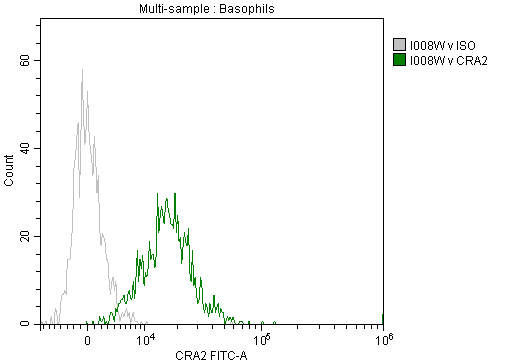


d

Negative control
Total FcεRI

Negative control
Unoccupied FcεRI

**Repository Figure 1b** - *Gating strategy total and unoccupied FcεRI*
As a blocking solution, we used Human immunoglobulin - IG VENA 50g/l (Kedrion Bolognana, Italy). For our FACS protocol, anti-human CD193-APC (5E8, BD. Ref. 558208) and anti-human CD123-PE (9F5, BD. Ref. 555644) for the APC and PE Channels were used. The FcεRI were stained with CRA1-BV421 (334624, BioLegend®; staining of total FcεRI) and CRA2-FITC (GTX00853, GeneTex©; staining of unoccupied FcεRI) antibodies. The CRA1 antibody stains all FcεRI whereas the CRA2 antibody stains the IgE unoccupied FcεRI. Isotype control antibodies were IgG2b-BV421 (MPC-11, Biolegend. Ref. 400307) and IgG1-FITC (R&D Systems, IC002F).
CytExpert© was used for analyzing the FACS data. The used Gain settings are stated here: FSC, SSC, CD63 (FITC), CD203c (PE), APC, PB450. The compensation matrix was not altered (values = 0,00) except for FITC-PE (1,00) and PB450-APC (1,50).
a) With FSC on the X-axis and SSC on the Y-axis, the basophil containing leukocytes were selected.
b) These selected leukocytes were then further divided by sorting them according to their CD193-APC and CD123-PE signals, marking the basophil population.
c,d) The median flourescence intensity (MFI) was calculated for CRA1 (total FceRI, magenta curve) and CRA2 (free FceRI, green curve) individually in each patient. In the histogram above, the negative control is visualized with the gray curve.


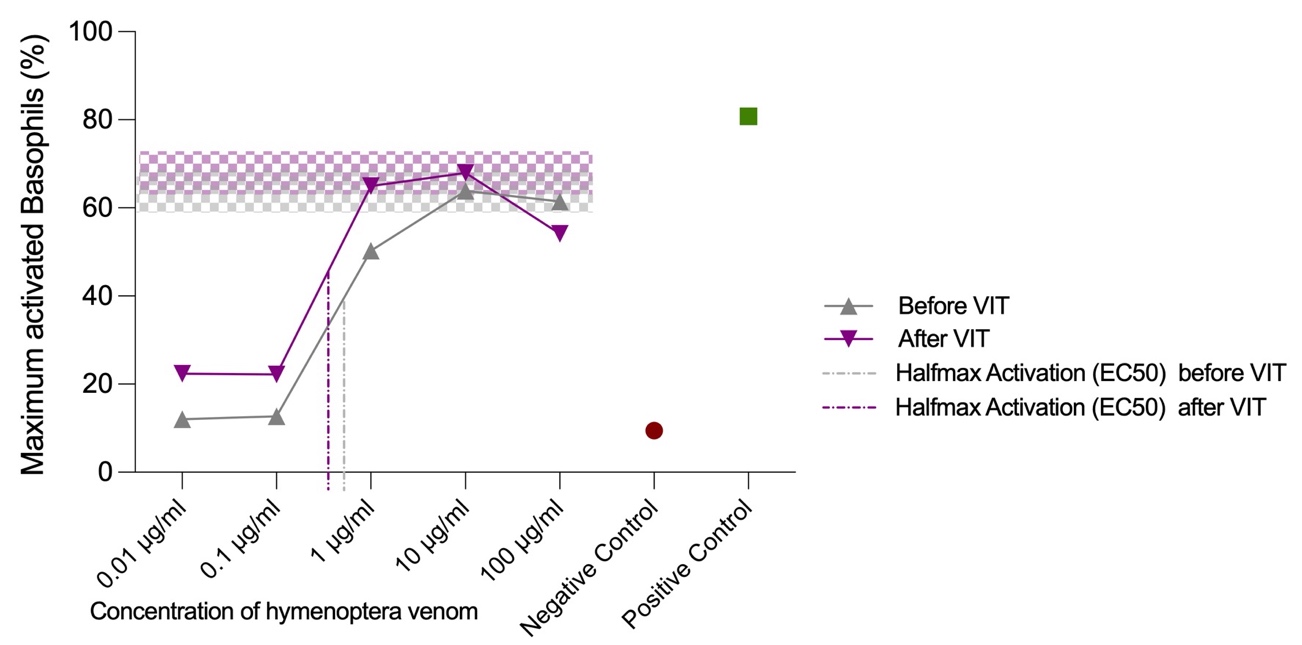


**Repository Figure 2** – Basophil activation test (BAT).
Maximum activated Basophils at certain concentration steps including positive and negative control.
Dashed vertical line indicates calculated EC50. Shaded Background indicates 10 % range of max. activation.

A positive and negative control was performed in each case. If the negative control of the BAT reached ≥21 % activation, the patient was excluded for further analysis due to high pre-activation. Furthermore, patients with a positive control of <40 % activation were excluded, because of lacking basophil response during BAT. Therefore, seven individuals were excluded in further statistical analysis with BAT.

The concentration at which maximum activation in the BAT was first achieved was determined with a 10 % range tolerance. This means that if a higher value was observed at a later ascending concentration of the insect toxin, but an earlier concentration already exhibited a percentage activation within 10 % of the previously mentioned maximum activation, this earlier value was considered the venom concentration (calculated concentration) at which the maximum activation was reached.

The EC50 in the manuscript is depicted as calculated concentration at which 50 % of the maximum activation of the basophils is reached, or as concentration step**/**range [<0.01 µg/ml, 0.1-0.01 µg/ml, 1-0.1µg/ml, 10-1 µg/ml, 100-10 µg/ml, 100 µg/ml (undiluted)] in which the respective calculated EC50 falls.

Abbreviations: VIT – venom immunotherapy. EC50 – effector concentration 50.


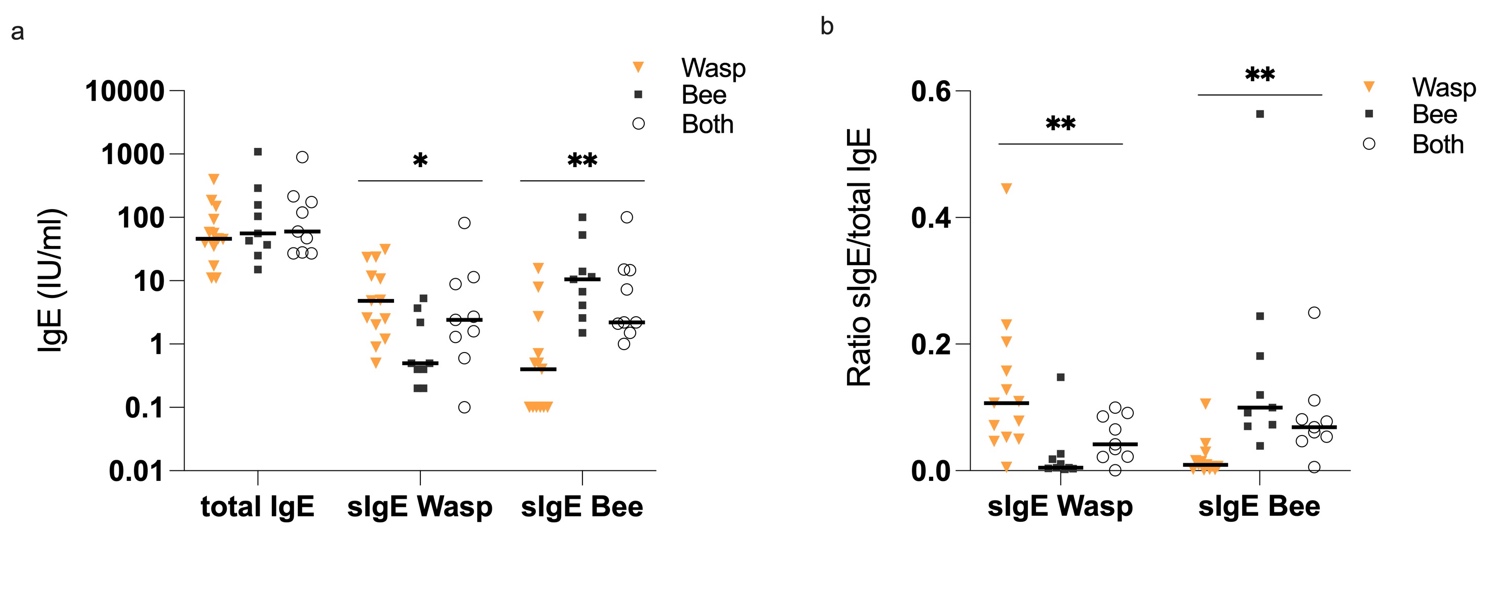

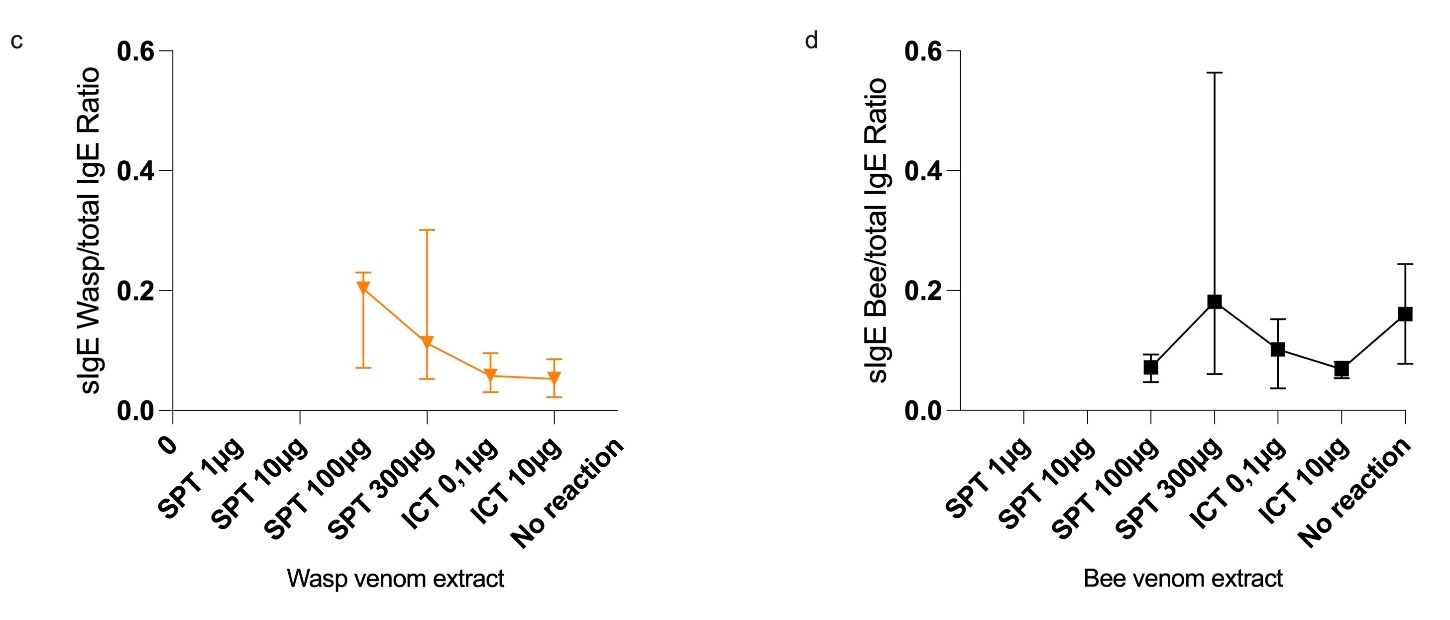


**Repository Figure 3**
a) Distribution of total and sIgE (extract) of wasp, bee and double sensitized patients. Horizontal bars represent median. Significances in Kruskal Wallis calculation are given as * p<0.05, **p<0.01.

b) Distribution of ratios of sIgE of venom extracts to total IgE of wasp, bee and double sensitized patients.
Horizontal bars represent median.
Significances between the groups in Kruskal Wallis calculation are given as * p<0.05, **p<0.01.

c+d) Correlation of ratios of sIgE of venom extracts to total IgE with the first positive Skin Prick Test (SPT)/ intracutaneous Test (ICT) test venom dilution Character indicates median, whiskers indicate IQR; positive if wheal was ≥3mm.

**
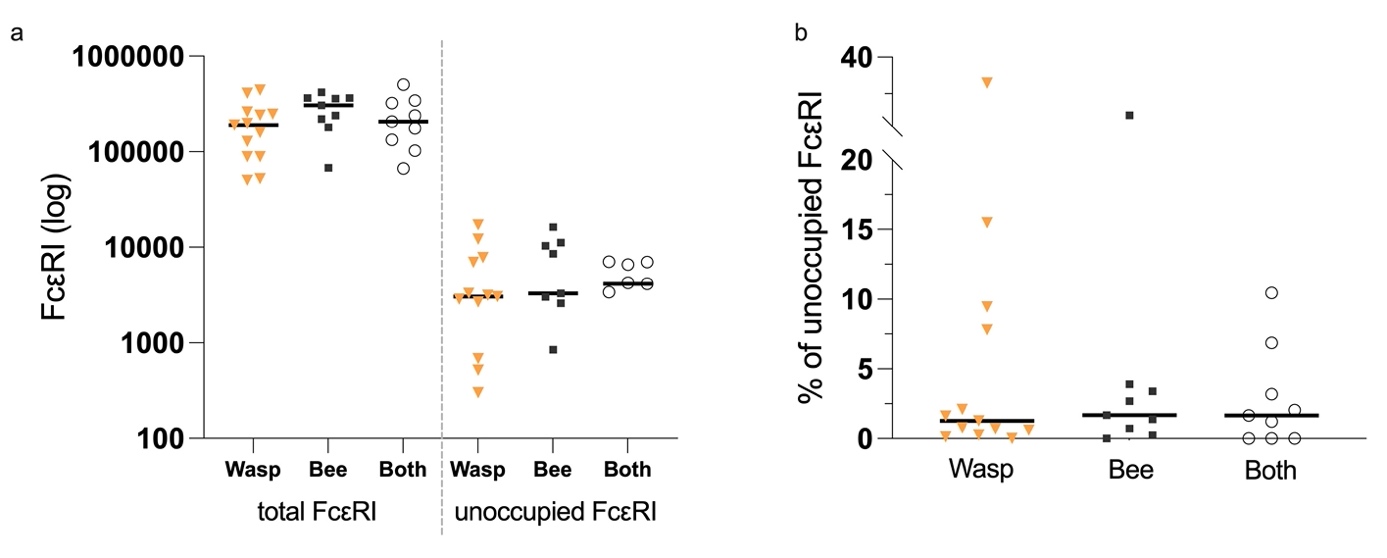
**

**Repository Figure 4** - Total and unoccupied FcεRI expression on basophils in HVA patients. FcεRI is shown as receptor per basophil.
a) Distribution of median FcεRI expression/basophil in the respective HVA patient group. In 1 wasp, 1 bee and 3 patients with both – no unoccupied FcεRI was detectable (values not depicted). Horizontal bars represent median values.
b) Percent of unoccupied FcεRI of total FcεRI are compared between the respective HVA group. Horizontal bars represent median values.


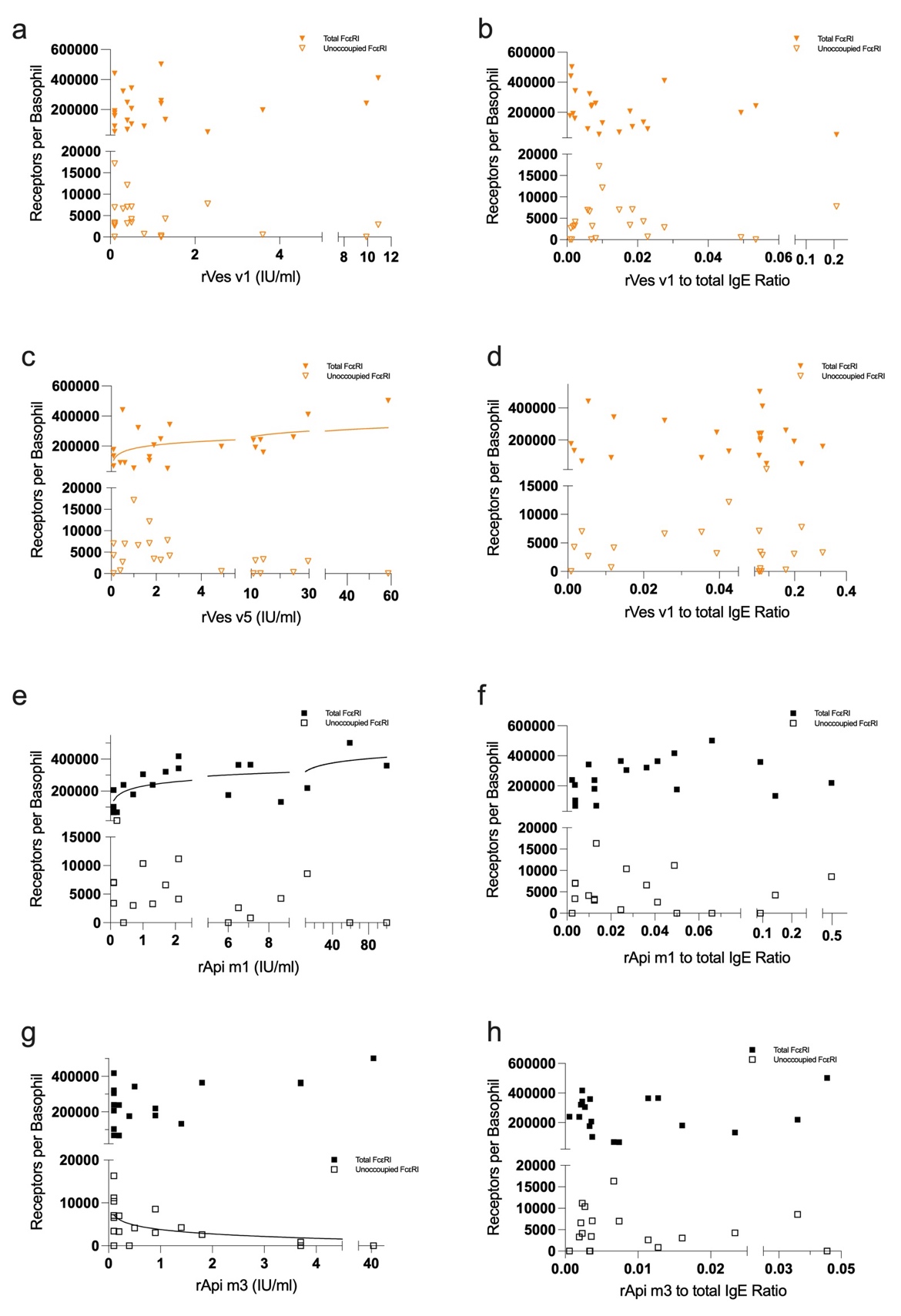


**Repository Figure 5** – Correlation of recombinant Allergens (and ratio with total IgE) with total and unoccupied FcεRI.
a +b) Recombinant Ves v1 and ratio to total IgE. b+c) Recombinant Ves v5 and ratio to total IgE. e+f) Recombinant Api m1 and ratio to total IgE. g+h) Recombinant Api m3 and ratio to total IgE. Significant values are indicated with non-linear fit (semi log) line.

**
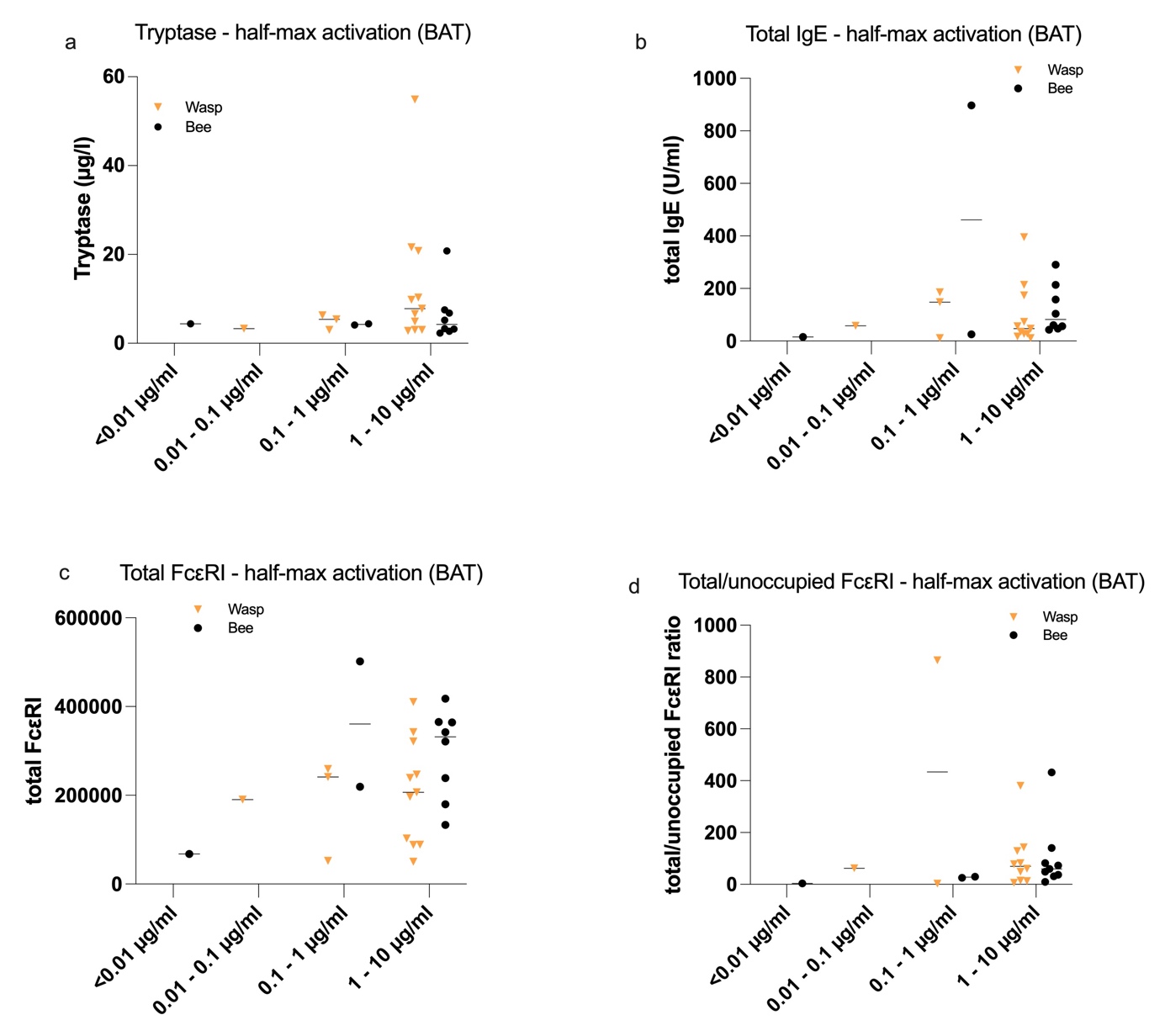
**

**Repository Figure 6** - Basophil activation test (EC50 step/range) with different laboratory values (a)Tryptase, b) total IgE, c) total FcεRI, d) ratio of total/unoccupied FcεRI)
